# A Rare twist: Case report of pediatric colo-colonic intussusception secondary to colonic schwannoma

**DOI:** 10.1016/j.ijscr.2025.111477

**Published:** 2025-06-06

**Authors:** Howard N. Rainey, Jed T. Ritter

**Affiliations:** Department of Emergency Medicine, Geisinger Medical Center, Danville, PA, USA

**Keywords:** Intussusception, Gastrointestinal schwannoma, Gastrointestinal autonomic nerve tumors

## Abstract

**Introduction and importance:**

Intussusception is a rare, but serious cause of abdominal pain that must be considered in pediatric emergency medicine, especially in younger patients.

**Case presentation:**

This report describes an 11-year-old male who presented to the emergency department (ED) with intermittent, waxing and waning abdominal pain, that later diagnosed as colo-colonic intussusception due to a colonic schwannoma lead point. Initial abdominal ultrasound revealed a complicated cystic at the descending colon. Follow-up computed tomography scan confirmed colo-colonic intussusception with a solid intra-luminal mass. Subsequent colonoscopy identified a five-centimeter broad based polypoid tumor at the splenic flexure which was non-resectable endoscopically. Left colon segmentectomy was performed removing six centimeters of bowel. Pathology diagnosed a benign colonic spindle schwannoma. Genetic screening was unremarkable, indicating a sporadic schwannoma that did not require additional surveillance or follow-up.

**Clinical discussion:**

Pediatric intussusception most commonly presents in individuals three months to six years of age, making the above individual outside of the typical presenting age. In addition, the lead point in this case, colonic schwannoma is incredibly rare, particularly in the pediatric population. The patient also had a colo-colonic intussusception which is not the typical location of pediatric intussusceptions.

**Conclusion:**

This case illustrates that, although most common in very young children, intussusception can occur at any age with the presence of a focal lead point and must remain on the differential diagnosis.

## Highlights

• Colonic schwannomas are rare, but possible, causes of pediatric intussusception
• Colo-colonic schwannomas are infrequently seen in the pediatric population and warrant more thorough workup
• Children with colonic schwannomas should raise suspicion for intussusception when presenting with abdominal pain

## 1 Introduction

Abdominal pain is one of the most frequent complaints seen in the ED among the pediatric population [1]. Although benign conditions such as viral gastroenteritis and constipation are more common causes of pediatric abdominal pain, it is crucial to rule out emergent etiologies in order to provide prompt intervention when necessary [2]. Among the most common emergent causes of pediatric abdominal pain is intussusception. Intussusception, which most commonly occurs in individuals between the ages of three months and six years, is a disease process in which the proximal bowel telescopes into the distal bowel, most commonly at the ileocolic region. Classically, this causes a triad of symptoms, including abdominal pain, emesis, and hematochezia. However, patients often present with non-specific symptoms that can make the diagnosis challenging [3]. Though there are several notable predisposing factors to pediatric intussusception, 90 % of cases are deemed idiopathic with no identifiable precipitating lesion [4]. In the remaining 10 %, a lead point is discovered. Practically speaking, any space-occupying lesion in the intestines can precipitate intussusception. Common causes include hypertrophied Peyer's patches, Meckel's diverticula, intestinal duplications, lymphoma, or polyps [[5], [6], [7]]. One rare condition that can cause intussusception is colonic schwannoma. This benign tumor is most often associated with a prior diagnosis of Von Recklinghausen's disease [8,9]. Before this report, colonic schwannoma was only identified in a single pediatric case of intussusception as the lead point [10]. In the following report, we present the clinical case of an 11-year-old male with a very rare colo-colonic intussusception secondary to a colonic schwannoma lead point. The below report is in-line with the SCARE criteria [11].

## 2 Case description

An otherwise healthy 11-year-old Caucasian male presented to the ED with his mother, complaining of progressively worsening, waxing and waning left lower quadrant abdominal pain that had been ongoing for approximately one week. The patient denied any associated symptoms except for nausea and a single episode of emesis that occurred just prior to arriving at the ED. Specifically, he denied fever, diarrhea, hematochezia, constipation, or urinary complaints. No prior personal or family history of gastrointestinal disease was reported. The patient's initial vital signs were remarkable only for mild tachycardia. His initial physical examination was also benign, with a notably reassuring abdominal examination.

**Fig. 1:**
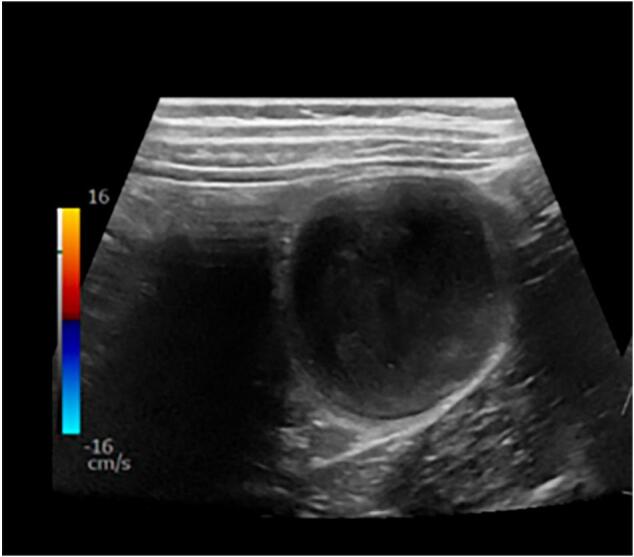
A complicated cystic structure at the left lower quadrant descending colon with peripheral vascularity measuring up to 4.4cm.

**Fig. 2:**
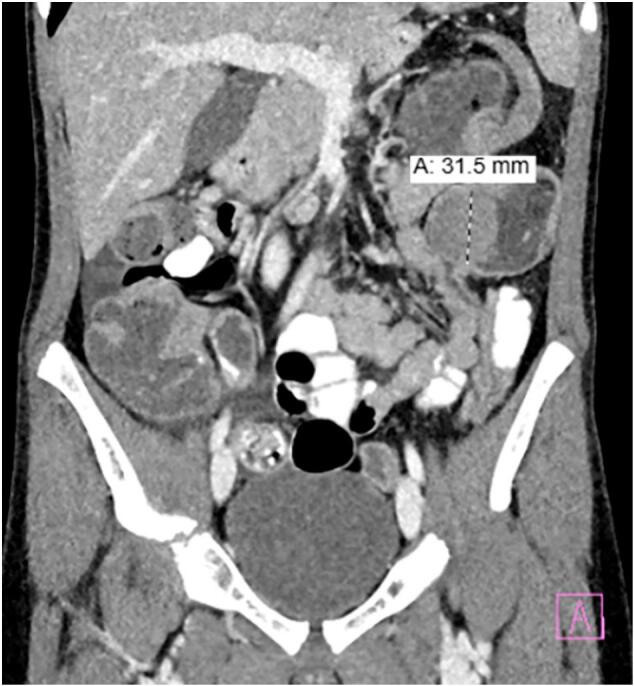
Colo-colonic intussusception with solid intraluminal mass.

Given his presentation, a series of laboratory tests were obtained, including complete blood count with differential, comprehensive metabolic panel, C-reactive protein, and urinalysis. The results were reassuring with no clear indication of the underlying cause of his symptoms. The patient was reassessed following lab testing, but his symptoms returned despite being asymptomatic on initial evaluation. He now had significant pain in his left lower quadrant on palpation. He was given acetaminophen for analgesia, and an abdominal ultrasound was promptly ordered. The ultrasound revealed a complicated cystic structure in the left lower quadrant at the descending colon with surrounding peripheral vascularity (Fig. 1). To better characterize this mass, computed tomography (CT) scan of the abdomen and pelvis with contrast was ordered at the recommendation of Pediatric Surgery, who consulted to assist in diagnosis and management. The CT scan revealed colo-colonic intussusception with a solid 4.8 × 4.5 × 3.3 cm intraluminal mass (Fig. 2). While in the ED, the patient's pain continued to worsen, necessitating intravenous opioids for adequate analgesia. Given the CT findings, Pediatric Surgery decided to admit the patient to the hospital for further management.

Upon admission to the hospital, Gastroenterology was consulted, and in agreement with Pediatric Surgery, recommended a colonoscopy to directly assess for intraluminal disease. The patient was managed symptomatically overnight and taken for colonoscopy the next morning. The procedure revealed a 5-cm broad based polypoid tumor of the splenic flexure. Unfortunately, the mass was unresectable via colonoscopy, so he was taken directly to the operating room. There, he underwent an open left colon segmentectomy with resulting 6-cm mass resection. There were no notable intraoperative complications. The specimen was immediately sent to pathology for evaluation. By post-operative day three, the patient was able to tolerate oral intake, resumed normal bowel function, and was deemed safe for discharge.

Approximately 10 days after his discharge, he had an outpatient follow-up appointment to discuss the pathology findings. The pathology report indicated a benign colonic spindle cell schwannoma. At that time, Pediatric Oncology was consulted and recommended a referral to medical genetics to evaluate for an underlying hereditary component, but no additional surveillance or intervention was deemed necessary. In follow-up, the patient was seen by genetics who recommended genetic screening, particularly for neurofibromatosis type two, with plans to analyze the LZTR1, NF2, and SMARCB1 genes. After thorough discussion, the patient and family elected to pursue the Invitae Hereditary Schwannomatosis panel. Fortunately, this screening was ultimately negative for any common hereditary pattern. Given this finding, the schwannoma was deemed sporadic, and the patient and family were counseled that further surveillance was likely unnecessary.

## 3 Discussion

Gastrointestinal autonomic nerve tumors (GANTs) are uncommon, representing approximately 0.1 % of benign tumors of the gastrointestinal tract [12,13]. One type of GANT is a schwannoma, which was first described in 1910 by Dr. Verocay. Although the identification of schwannomas has increased due to improved immunohistochemical staining techniques, they remain quite rare [14]. Gastrointestinal schwannomas are derived from nerve sheath cells, or Schwann cells [15]. Among the types of gastrointestinal schwannomas, colorectal schwannomas are the most uncommon [16]. These tumors are largely asymptomatic, though non-specific symptoms such as fatigue, abdominal pain, rectal bleeding, changes in bowel patterns, obstruction, or tenesmus can occur [17]. A case of intussusception secondary to an intestinal schwannoma has been described in the literature, though this is a rare occurrence, with only one such incidence reported to date [10].

Gastrointestinal schwannomas make up approximately 2.0–6.0 % of all mesenchymal tumors [18]. Most commonly, gastrointestinal schwannomas arise from the stomach (70 %), but they have also been reported in the colon, rectum, small intestine, esophagus, and pancreas [19]. These lesions are most common in females in the fifth to sixth decade of life and are very seldomly seen in younger populations [20]. When schwannomas are identified after resection, follow-up genetic screening is often crucial due to their associations with neurofibromatosis type 1, or von Recklinghausen's disease [21]. Genetic screening is important when schwannomas are discovered as the patient would be at further risk for additional tumors elsewhere and would require lifetime surveillance.

As discussed, intussusception is a rare but important consideration in patients presenting to the ED with abdominal pain. Intussusception is primarily a disease of young children, with most cases occurring between three months and six years of age [22,24]. This condition has also been noted in adulthood, typically due to a mass lesion, such as a schwannoma. Clinically significant intussusception is most often found at the ileocolic junction but can occur throughout the bowel. Most cases of intussusception are idiopathic, with only around 10 % having a notable lead point. Among these lead points, gastrointestinal schwannomas are possible, though quite rare, particularly in children.

Prior to our case above, only one identified case of intussusception caused by a gastrointestinal schwannoma lead point had been described in the pediatric setting. The presented case exhibits multiple rarities that make it particularly interesting and highlight the importance of considering intussusception even during atypical circumstances. The patient's age is quite uncommon for both intussusception and colonic schwannoma. Intussusception typically presents between three months and six years of age, while colonic schwannomas are almost exclusively seen in individuals in their fifth to sixth decade of life. Interestingly, our patient was 11 years old, clearly outside of the common age range for both conditions. Additionally, the patient had a colo-colonic intussusception which is rare in general and particularly in pediatrics, where ileocolic intussusception is more common. Finally, colonic schwannomas are often associated with von Recklinghausen's disease [23]. This patient underwent genetic screening after tumor resection, which was unremarkable for all markers associated with NF1. This highlights that these tumors can be present without an underlying genetic condition, and emergency clinicians should not exclude the diagnosis based on the absence of a predisposing condition.

## 4 Conclusion

This case underscores the critical need for emergency clinicians to consider intussusception in their differential diagnosis, regardless of age or typical predisposing conditions to ensure timely and accurate intervention.

## Consent

Written informed consent was obtained from the patient for publication of this case report and accompanying images. A copy of the written consent is available for review by the Editor-in-Chief of this journal on request.

## Ethical approval

Case Report is exempt from ethical approval as this is a case report without any patient identifiers.

## Funding

No reportable sources of funding.

## Author contribution

Drs. Howard Rainey and Jed Ritter had equal contribution to the construction and editing of this report.

## Guarantor

Howard N Rainey.

## Research registration number

N/A.

## Conflict of interest statement

We have no conflicts of interest to report.
